# Decreasing serum 25‐hydroxyvitamin D levels and risk of early neurological deterioration in patients with ischemic stroke

**DOI:** 10.1002/brb3.1227

**Published:** 2019-02-06

**Authors:** Wei Hu, Dezhi Liu, Qin Li, Li Wang, Qiqiang Tang, Guoping Wang

**Affiliations:** ^1^ Department of Neurology, The First Affiliated Hospital of USTC Division of Life Science and Medicine University of Science and Technology of China HeFei Anhui China; ^2^ Department of Neurology Shanghai General Hospital Shanghai Jiao Tong University School of Medicine Shanghai P.R. China; ^3^ The Central Laboratory of Medical Research Center The First Affiliated Hospital of USTC, Division of Life Science and Medicine University of Science and Technology of China HeFei Anhui China

**Keywords:** 25‐hydroxyvitamin D, early neurological deterioration, ischemic stroke

## Abstract

**Background and Aims:**

Vitamin D deficiency has been linked to a higher risk of ischemic stroke. We therefore explored the relationship between serum 25‐hydroxyvitamin D [25(OH)D] levels and early neurological deterioration (END) after acute ischemic stroke in a hospital‐based prospective study.

**Methods:**

From June 2016 to June 2018, patients with ischemic stroke within 48 hr from symptom onset were consecutively recruited. Serum 25(OH)D levels were measured at admission. END was defined as an increase of ≥1 point in motor power or ≥2 points in the total National Institute of Health Stroke Scale score within 7 days after admission. Multiple logistic regression models were performed to calculate the odds ratio (OR) and confidence intervals (CI) of 25(OH)D levels in predicting END.

**Results:**

A total of 478 subjects were enrolled, of which 136 (28.5%) patients developed END. The mean 25(OH)D levels were 49.5 ± 15.8 nmol/L. Univariate logistic regression analysis showed that advanced age, white matter lesions, high level of body mass index, diastolic blood pressure, fasting blood glucose and homocysteine, and low 25(OH)D levels were associated with END. Furthermore, multivariate regression analysis demonstrated that the first quartile of 25(OH)D concentrations [OR, 2.628; 95% CI,1.223–5.644; *p* = 0.013] was independently risk factor for END.

**Conclusions:**

This study illustrated that lower 25(OH)D levels might be associated with an increasing risk of END in acute ischemic stroke patients.

## 1 INTRODUCTION

Stroke is the leading cause of mortality and permanent disability, contributing to a tremendous burden on health resources in China (Liu et al., 2007). Although most ischemic stroke patients tend to improve over the subsequent few days after symptoms onset, a sizeable fraction does not substantially recover but even deteriorates, which has been identified as early neurological deterioration (END) (Seners, Turc, Oppenheim, & Baron, 2015). Previous studies reported that END after acute ischemic stroke is observed in 5%–40% patients and often leads to an increasing risk of functional disability and mortality (Helleberg, Ellekjær, Rohweder, & Indredavik, 2014; Kwon, Lee, Bae, & Kang, 2014; Seners et al., 2015; Sun et al., 2014; Zhang et al., 2016). Accordingly, more precise understanding of the potential mechanisms involved in END could provide valuable insights for improving stroke outcomes.

Vitamin D is recognized as a hormone that mainly regulates calcium metabolism, and possesses a strong anti‐inflammatory effect (Buell & Dawson‐Hughes, 2008). The inactive vitamin D is hydroxylated in the liver to form 25‐hydroxyvitamin D [25(OH)D], which is widely viewed as the superior biomarker for assessing vitamin D status (Hossein‐nezhad & Holick, 2012). Nowadays, vitamin D deficiency has been implicated as a potentially modifiable risk factor for ischemic cerebrovascular disease. A population‐based study nested in Hong Kong Chinese indicated that lower 25(OH)D levels might increase the risk of incident ischemic stroke (Leung et al., 2017). Recently, several studies have reported the significant correlation between 25(OH)D status and functional outcome after acute ischemic stroke (Park et al., 2015; Tu, Zhao, Xu, & Chen, 2014; Wang, Ji, Tong, & Zhang, 2014). Nevertheless, whether 25(OH)D levels are associated with acute ischemic stroke complications, such as END, has not been well clarified. We therefore performed this prospective study to evaluate the relationship between 25(OH)D levels and the development of END in patients with acute ischemic stroke.

## 2 MATERIALS AND METHODS

### 2.1 Subjects

This was a hospital‐based prospective study screening patients in Anhui Provincial Hospital from June 2016 to June 2018. Patients diagnosed as first‐ever ischemic stroke and hospitalized within 48 hr of symptoms onset were initially considered for inclusion in this study. Patients with age less than 18 years old, renal insufficiency, severe hepatic disease, severe heart failure, malignant tumor, acute or chronic inflammatory disease, autoimmune diseases, and early discharge were not included. We also excluded those received intravenous thrombolysis or an emergent endovascular procedure. This study was approved by the institutional review board of Anhui Provincial Hospital. Informed consent was obtained from participants or legal representatives.

### 2.2 Clinical data collection

Demographic characteristics and clinical data were collected at the time of enrollment. Variables were recorded as follows: (a) demographic characteristics, hypertension, diabetes mellitus, hyperlipidemia, current smoking and drinking, atrial fibrillation and medical history; (b) clinical parameters including systolic and diastolic blood pressure level, body mass index, stroke severity and stroke subtypes. Stroke subtypes was classified according to TOAST (Trial of Org 10172 in Acute Stroke Treatment) criteria (Ir, 1999). Stroke severity was assessed using the National Institutes of Health Stroke Scale (NIHSS) score. Functional outcome was evaluated at discharge using the modified Rankin scale. We also performed laboratory tests for hyper‐sensitive C‐reactive protein (immunoturbidimetry), fasting blood glucose (glucose oxidase assay), lipid profile (ALBK assay), homocysteine (immunoturbidimetry), and 25(OH)D level (chemiluminescent immunoassay).

### 2.3 Definition of END

Stroke severity was assessed using NIHSS at admission and continued 1–3 times a day within the 7 days after admission. The neurologist who performed serial neurologic examinations was certified in the NIHSS scoring and blinded to clinical information. In accordance with previous studies, we collected END cases using the following criteria: an increment of ≥1 point in motor power or ≥2 points in the total NIHSS score within 7 days (Kwon, Lim, Park, & Lee, 2011; Kwon et al., 2014; Zhang et al., 2016).

### 2.4 Measurement of serum 25(OH)D

Blood sample was collected from all patients within 24 hr after admission. After at least 30 min of clotting, the specimens were centrifuged at 1500 g for 10 min and the isolated serum were stored at −80°C until analysis. The 25(OH)D levels were measured by chemiluminescent immunoassay (Siemens Healthcare Diagnostics, Inc, NY). The intra‐ and inter‐assay coefficients of variation were 5.1% and 4.4%, respectively, with a standard concentration in these kits ranging from 10 to 275 nmol/L. All procedures were conducted in strict accordance to manufacturers' instructions.

### 2.5 Statistical analysis

All statistical analyses were performed using SPSS version 23.0 for Windows (SPSS IBM Inc, Chicago, IL). Continuous variables were presented as the means (standard deviation, *SD*) or median (interquartile range), and categorical variables were expressed as *n* (%). Differences in baseline characteristics according to quartile of 25(OH)D concentrations were determined using χ^2^, analysis of variance, or Kruskal–Wallis where appropriate. Binary logistic regression analysis was used to assess the association between 25(OH)D levels and END. Multivariable analysis was adjusted for the factors with a *p* < 0.1 in the univariable analysis (including age, white matter lesions, body mass index, diastolic blood pressure, fasting blood glucose and homocysteine levels). Kaplan–Meier curves were performed to estimate the probability of END stratified by 25(OH)D quartile. We also used the receiver operating characteristic (ROC) curves to measure the diagnostic performance of 25(OH)D concentrations. All tests were two‐tailed and statistical significance was established at *p* < 0.05.

## 3 RESULTS

**Figure 1:**
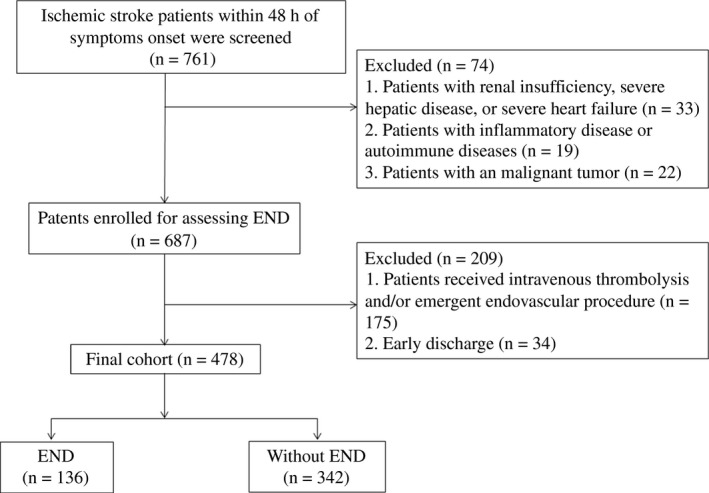
Flowchart of patient inclusion

We recruited 478 patients for the final analysis (Figure 1). Mean age of patients was 62.8 years (*SD* 9.9) and 52.3% were men. Among these patients, 66.1% had hypertension, 22.0% had diabetes mellitus and 16.9% had hyperlipidemia. The mean 25(OH)D levels were 49.5 ± 15.8 nmol/L, with quartile level as follows: first quartile (<40.5 nmol/L), second quartile (40.5–51.9 nmol/L), third quartile (52.0–60.4 nmol/L), and fourth quartile (>60.4 nmol/L).

Table 1 demonstrated the main demographic, clinical and imaging characteristics of the study population stratified by the 25(OH)D quartile. In general, decreased 25(OH)D level showed a significant correlation with age (*p *=* *0.014), female sex (*p *=* *0.003), diabetes mellitus (*p *=* *0.006), white matter lesions (*p *=* *0.001), END (*p *=* *0.001), died at discharge (*p *=* *0.042), body mass index (*p *=* *0.028), systolic blood pressure (*p *=* *0.002), fasting blood glucose (*p *=* *0.027) and total cholesterol level (*p *=* *0.027). However, there was no significant difference between 25(OH)D levels and NIHSS score (*p *=* *0.339), and stroke subtypes (*p *=* *0.293).

**Table 1:** Baseline characteristics of the study population stratified by the 25(OH)D quartile

Variable	25(OH)D quartile				*p* value
	First (*n* = 118)	Second (*n* = 124)	Third (*n* = 120)	Fourth (*n* = 116)	
Demographic characteristics					
Age, year	65.8 ± 11.0	63.1 ± 10.8	61.8 ± 8.9	60.4 ± 8.1	0.014
Female, %	60 (50.8)	76 (61.3)	40 (33.3)	52 (44.8)	0.003
Vascular risk factors, %					
Hypertension	84 (71.2)	88 (71.0)	76 (63.3)	68 (58.6)	0.113
Diabetes mellitus	36 (30.5)	31 (25.0)	24 (20.0)	14 (12.1)	0.006
Hyperlipidemia	24 (20.3)	27 (21.8)	14 (11.7)	16 (13.8)	0.102
Current drinking	22 (18.6)	29 (23.4)	30 (25.0)	28 (24.1)	0.658
Current smoking	33 (27.9)	44 (35.4)	37 (30.8)	29 (25.0)	0.330
Atrial fibrillation	20 (16.9)	18 (14.5)	14 (11.7)	20 (17.2)	0.600
Clinical data					
Previous antiplatelet, %	22 (18.6)	26 (20.9)	16 (13.3)	20 (17.2)	0.461
Previous statin, %	11 (9.3)	14 (11.3)	10 (8.3)	14 (12.1)	0.784
Body mass index, kg/m^2^	25.3 ± 3.4	24.8 ± 3.8	24.8 ± 3.1	23.8 ± 2.6	0.028
Systolic blood pressure, mmHg	141.8 ± 22.6	138.8 ± 17.9	136.1 ± 17.6	133.0 ± 15.6	0.002
Diastolic blood pressure, mmHg	82.2 ± 9.9	81.5 ± 11.3	80.4 ± 10.5	81.6 ± 10.7	0.627
Silent lacunar infarction, %	50 (42.4)	48 (38.7)	53 (45.7)	47 (37.3)	0.308
White matter lesions, %	52 (44.1)	46 (37.1)	30 (25.0)	20 (17.2)	0.001
NIHSS, score	4.0 (2.0, 8.0)	4.0 (2.0, 8.5)	4.0 (2.0, 6.0)	4.0 (2.0, 7.0)	0.339
END, %	44 (37.3)	42 (33.9)	32 (26.7)	18 (15.5)	0.001
Died at discharge, %	17 (14.4)	18 (14.5)	6 (5.0)	10 (8.6)	0.042
mRS at discharge, %					0.056
0‐2	63 (53.4)	74 (59.7)	83 (69.2)	77 (66.4)	
3‐6	55 (46.6)	50 (40.3)	37 (30.8)	39 (33.6)	
Stroke subtype, %					0.293
Large artery atherosclerosis	41 (34.7)	50 (40.3)	47 (39.2)	31 (26.7)	
Cardioembolism	22 (18.6)	20 (16.1)	20 (16.7)	23 (19.8)	
Small artery occlusion	36 (30.5)	41 (33.1)	34 (28.3)	50 (43.1)	
Other determined etiology	9 (7.6)	6 (4.8)	5 (4.2)	5 (4.3)	
Undetermined etiology	10 (8.5)	7 (5.6)	14 (11.7)	7 (6.0)	
Laboratory data					
Total cholesterol, mmol/L	4.7 ± 1.1	4.5 ± 0.9	4.2 ± 1.1	4.2 ± 1.3	0.027
Triglyceride, mmol/L	1.3 (1.0, 1.8)	1.3 (1.0, 2.0)	1.2 (1.0, 1.6)	1.4 (0.9, 2.1)	0.476
High‐density lipoprotein, mmol/L	1.3 (1.2, 1.6)	1.3(1.1, 1.5)	1.2 (1.1, 1.5)	1.4 (1.2, 1.6)	0.243
Low‐density lipoprotein, mmol/L	2.5 ± 1.0	2.5 ± 0.8	2.4 ± 0.7	2.7 ± 1.0	0.229
Hs‐CRP, mg/L	3.0 (1.0, 6.0)	4.0 (1.0, 8.0)	3.0 (1.0, 6.5)	3.0 (1.0, 5.8)	0.108
Fasting blood glucose, mmol/L	6.4 ± 2.1	6.1 ± 1.9	5.8 ± 1.7	5.8 ± 1.8	0.027
Homocysteine, mmol/L	14.1 ± 9.7	14.7 ± 8.6	13.4 ± 5.4	13.3 ± 4.8	0.532

*Note*. 25(OH)D: 25‐hydroxyvitamin D; END: early neurological deterioration; Hs‐CRP: hyper‐sensitive C‐reactive protein; mRS: modified Rankin scale; NIHSS: national institute of health stroke scale.

**Figure 2:**
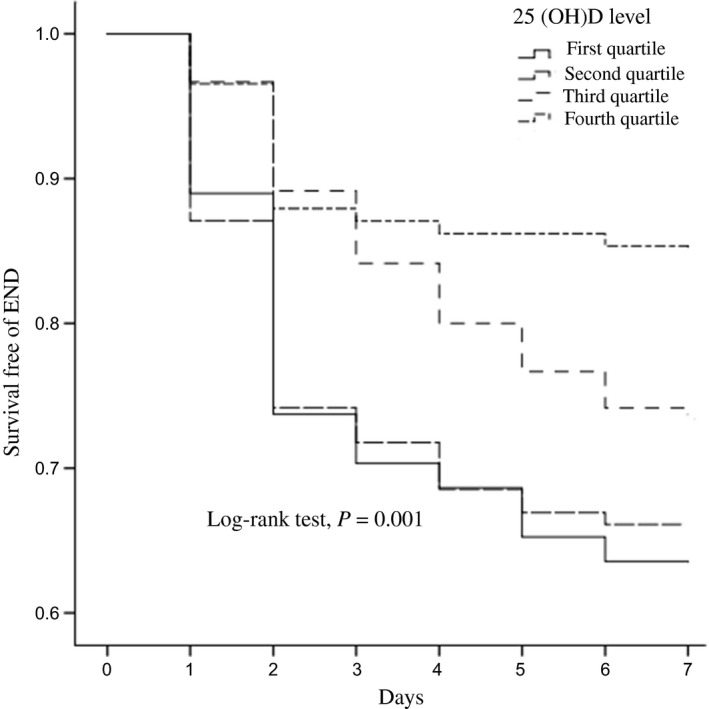
Kaplan–Meier curves estimates the probability of END stratified by 25(OH)D quartiles. 25(OH)D indicates 25‐hydroxyvitamin D; and END, early neurological deterioration

END was observed in 136 patients, which accounted for 28.5% [95% confidence interval (CI) 25.6%–31.4%] of the cohort. As shown in Figure 2, patients with lower 25(OH)D levels portended an increasing risk of END, which mostly developed within the 48 hr after admission. Table 2 summarized the results of the binary logistic regression of the END. Univariate logistic regression analysis showed that advanced age, white matter lesions, high level of body mass index, diastolic blood pressure, fasting blood glucose and homocysteine, and lower 25(OH)D levels were associated with END. Furthermore, after controlling for potential confounders, the first quartile of 25(OH)D concentrations [odds ratio (OR), 2.622; 95% CI, 1.226–5.641; *p *=* *0.015] was an independent risk factor for END.

**Table 2:** Univariate and multivariate logistic regression analysis for END

	Univariate analysis		Multivariate analysis	
	OR (95% CI)	*p*	OR (95% CI)	*p*
Select variables				
Age	1.024 (1.008–1.142)	0.044		
Female	1.322 (0.901–1.996)	0.148		
Hypertension	0.801 (0.529–1.212)	0.294		
Diabetes mellitus	1.069 (0.664–1.721)	0.783		
Hyperlipidemia	0.676 (0.383–1.190)	0.175		
Current drinking	1.059 (0.622–1.695)	0.811		
Current smoking	1.353 (0.885–2.070)	0.163		
Atrial fibrillation	0.962 (0.550–1.681)	0.891		
Previous antiplatelet	1.157 (0.693–1.932)	0.576		
Previous statin	1.123 (0.590–2.136)	0.724		
Body mass index	1.083 (1.019–1.151)	0.012		
Systolic blood pressure	1.007 (0.996–1.017)	0.201		
Diastolic blood pressure	1.019 (1.000–1.038)	0.049		
NIHSS score	1.027 (0.986–1.069)	0.203		
Silent lacunar infarction	1.206 (0.811–1.796)	0.355		
White matter lesions	1.737 (1.144–2.638)	0.010		
Hs‐CRP	1.004 (0.980–1.020)	0.992		
Fasting blood glucose	1.117 (1.011–1.234)	0.030		
Homocysteine	1.037 (1.007–1.067)	0.016		
25(OH)D level				
As continuous variable	0.976 (0.961–0.987)	0.001	0.973 (0.958–0.991)	0.005
As categorical variable				
Fourth quartile	Reference		Reference	
Third quartile	1.248 (0.938–3.774)	0.108	1.188 (0.540–2.615)	0.664
Second quartile	2.789 (1.492–5.211)	0.006	1.925 (0.922–4.021)	0.081
First quartile	3.237 (1.731–6.054)	0.001	2.622 (1.226–5.641)	0.015

*Notes*. 25(OH)D: 25‐hydroxyvitamin D; CI: confidence interval; END: early neurological deterioration; Hs‐CRP: hyper‐sensitive C‐reactive protein; NIHSS: national institute of health stroke scale; OR: odds ratio.

Multivariate analysis adjusted for age, sex, white matter lesions, body mass index, diastolic blood pressure, fasting blood glucose and homocysteine level.

The ROC curves showed the optimal cutoff value of serum 25(OH)D levels as an END indicator was estimated to be 42.5 nmol/L, which yielded a sensitivity of 71.9% and a specificity of 60.3%, with the area under curve of 0.635 (95% CI 0.580–0.689).

## 4 DISCUSSION

In this hospital‐based prospective study, END was observed in 136 (28.5%) patients, which is similar to previous data ranging from 5% to 40% (Kwon et al., 2014; Seners et al., 2015; Sun et al., 2014; Zhang et al., 2016). We also found that lower 25(OH)D serum levels were associated with the presence of END in patients with acute ischemic stroke. This association was independent of other well‐known predictors of neurological worsening such as age, cardiovascular risk factors, and neurological severity on admission.

Vitamin D refers to a group of fat‐soluble secosteroids hormones, and is typically ingested in dietary sources or produced in the skin as a result of sunlight exposure (Holick, 2007; Hossein‐nezhad & Holick, 2012). Its serum level is significantly decreased in various chronic diseases. In humans, vitamin D deficiency is considered to be related with the following disorders: hypertension, diabetes mellitus, metabolic syndrome, arterial stiffening, left ventricular hypertrophy, vascular dysfunction, and renin‐angiotensin system activation (Al Mheid & Quyyumi, 2017; Michos & Melamed, 2008). Over the past few years, accumulating epidemiological evidence illustrated that decreased 25(OH)D levels have been linked to an increased risk of coronary heart disease, cognitive decline and ischemic stroke (Balion et al., 2012; Giovannucci, Liu, Hollis, & Rimm, 2008; Poole et al., 2006). Currently, several studies have focused on the association between vitamin D levels with functional outcomes after ischemic stroke. In previous studies based on Caucasian stroke population, low 25(OH)D levels have been reported to be an indicator of unfavorable functional outcome at discharge (OR 2.06; 95% CI 1.06–3.94, *p *=* *0.03) and 1‐year mortality (Harzad Ratio 1.95; 95% CI 1.14–3.32, *p *=* *0.014) (Daubail et al., 2013, 2014). Furthermore, serum 25(OH)D level was a predictor of both severity at admission (*r* = −0.363, *p *<* *0.001) and functional outcome at discharge (OR 0.79; 95% CI 0.73–0.85; *p *=* *0.005) in a cohort of Chinese ischemic stroke patients (Wang et al., 2014). However, we did not detect an apparent association between 25(OH)D levels and stroke severity (*r* = −0.056, *p *=* *0.225). This discrepancy might be explained at least in part by differences concerning the study design, especially the study population and sample size. Furthermore, on the contrary to previous results (Wat, Leung, Tam, & Kung, 2007), our study found that female have a higher prevalence of vitamin D deficiency than male. Nevertheless, this hypovitaminosis D does not appear to be associated with increased END risk among female. Future clinical studies with large sample sizes and multicenter data are needed to confirm our findings.

Emerging data from large clinical trials have suggested that vitamin D may be important for neurovascular protection. Thus far, no precise data are available regarding the association of Vitamin D deficiency with END. Serum 25(OH)D concentrations were reported to be associated with blood pressure level, glucose and lipid metabolism, which may exacerbate the development of END (Alvarez‐Sabín et al., 2003; Buell & Dawson‐Hughes, 2008; Pezzini et al., 2011). In our study, we performed a fully adjusted model after adjusting for age, sex, diastolic blood pressure and fasting blood glucose, and found that the association between 25(OH)D levels and risk of END remained robust. Several plausible biological mechanisms might be proposed for this relationship between the 25(OH)D levels and neurological worsening during the acute phase of stroke. There is a growing body of evidence that vitamin D possesses an anti‐inflammatory property and vitamin D deficiency may contribute to overall increased inflammatory activity (Takeda et al., 2010; Wong, Man, & Vanhoutte, 2010). In return, chronic inflammation is a pathological condition characterized by tissue destruction, promotes oxidative stress and attenuates cellular antioxidant capacity, which may propagate brain damage after acute stroke (Cruz‐Álvarez et al., 2017; Vila, Castillo, Dávalos, & Chamorro, 2000; Vila et al., 2003). In a rat model of local cerebral ischemia induced by ligation of the middle cerebral artery, 1,25(OH)2D3 administration was correlated to a significant reduction in brain ischemic infarct size (Wang et al., 2000). Also, rat models fed with a vitamin D‐deficient diet had higher risk of infarct volume growth compared with the controls (Balden, Selvamani, & Sohrabji, 2012). As infarct volume growth has been proposed as the main cause of neurological deterioration (Seners et al., 2015; Terasawa et al., 2008), we further hypothesized that the association of lower 25(OH)D levels with an increased risk of END may be partially explained by vitamin D deficiency‐induced inflammatory neuronal death and infarct volume increasing. Unfortunately, the infarct volume growth was not evaluated in our study, and consequently, we were not able to confirm this assumption.

Strengths of our study include using a standardized research method, large study size and the availability of a large array of information for covariate selection. However, some limitations of this observational study need to be discussed. First, the cross‐sectional design of our analyses limits our ability to determine a causal relationship between 25(OH)D levels and END. Second, 25(OH)D levels were only measured at admission, which yielded no data regarding the change of level in ischemic stroke patients. Third, the data on Vitamin D receptor gene polymorphisms, vitamin D supplementation, outdoor physical activity, sun exposure, health education, social level, and parathyroid hormone level were not collected in our study. Therefore, we could not adjust these variables in multivariable analysis, although their role in the regulating 25(OH)D levels has not been elucidated yet. In addition, the study was performed in one center with Chinese population, which might not be generalizable to other ethnic populations.

In conclusion, our study showed that lower 25(OH)D levels might be associated with a higher risk of END developing amongst acute ischemic stroke patients. Future randomized clinical controlled trials are therefore urgently needed to assess whether vitamin D supplementation could prevent neurological deterioration in the acute phase of ischemic stroke.

## DISCLOSURES

The authors have no financial conflicts of interest.
